# Horticultural Plant Residues as New Source for Lignocellulose Nanofibers Isolation: Application on the Recycling Paperboard Process

**DOI:** 10.3390/molecules25143275

**Published:** 2020-07-18

**Authors:** Isabel Bascón-Villegas, Eduardo Espinosa, Rafael Sánchez, Quim Tarrés, Fernando Pérez-Rodríguez, Alejandro Rodríguez

**Affiliations:** 1 Chemical Engineering Department, Bioagres group, Universidad de Córdoba, 14014 Córdoba, Spain; a.rodriguez@uco.es; 2 Department of Food Science and Technology, Universidad de Córdoba, 14014 Córdoba, Spain; b42perof@uco.es; 3 Technological Institute of Packaging, Transport and Logistic (ITENE), 46980 Paterna, Spain; rafael.sanchez@itene.com; 4 Group LEPAMAP, Department of Chemical Engineering, Universidad de Girona, 17071 Girona, Spain; joaquimagusti.tarres@udg.edu

**Keywords:** lignocellulose nanofibers, horticultural residues, paperboard, recycling

## Abstract

Horticultural plant residues (tomato, pepper, and eggplant) were identified as new sources for lignocellulose nanofibers (LCNF). Cellulosic pulp was obtained from the different plant residues using an environmentally friendly process, energy-sustainable, simple, and with low-chemical reagent consumption. The chemical composition of the obtained pulps was analyzed in order to study its influence in the nanofibrillation process. Cellulosic fibers were subjected to two different pretreatments, mechanical and TEMPO(2,2,6,6-Tetramethyl-piperidin-1-oxyl)-mediated oxidation, followed by high-pressure homogenization to produce different lignocellulose nanofibers. Then, LCNF were deeply characterized in terms of nanofibrillation yield, cationic demand, carboxyl content, morphology, crystallinity, and thermal stability. The suitability of each raw material to produce lignocellulose nanofibers was analyzed from the point of view of each pretreatment. TEMPO-mediated oxidation was identified as a more effective pretreatment to produce LCNF, however, it produces a decrease in the thermal stability of the LCNF. The different LCNF were added as reinforcing agent on recycled paperboard and compared with the improving produced by the industrial mechanical beating. The analysis of the papersheets’ mechanical properties shows that the addition of LCNF as a reinforcing agent in the paperboard recycling process is a viable alternative to mechanical beating, achieving greater reinforcing effect and increasing the products’ life cycles.

Academic Editor: Fabrizio Sarasini

## 1. Introduction

The 21st century industrial revolution boosted changes in methods of production and consumption thanks to several factors, such as technological development, globalization of markets and resources, availability of energy, etc. In terms of development and welfare, the linear economic system had been beneficial. However, this model, based on taking, making, and discarding [1], is not compatible with the limited resources and capacity to adapt to environmental impact [2]. As a consequence, nowadays, modern societies are affected by an alarming resource depletion and overproduction of materials which are very difficult to manage. As an alternative, it is proposed that the system takes a regenerative approach, using “the circular economy” concept, with the aim of keeping the resource value, and restricting the raw materials and energy inputs. This is generally known as the “bioeconomy”.

The bioeconomy is an economic system in which biomass is converted into value-added materials [3], such as chemicals [4], food and feed, and fuels and energy [5,6], among others. It is based on two assumptions: (i) The biomass is not being totally exploited, its main destination being soil protection, animal feed, burning, composting and silage; and (ii) the use of the biomass could be improved. This can be attained by a product valorization and a more efficient use of the agricultural residues mentioned, extracting more energy and byproducts from them, and decreasing both the cost of agricultural production and the spread of pests and greenhouse gas (GHG) emissions. In addition, the biomass application could be improved, increasing the process yields through innovative technological solutions [7].

The lignocellulosic biomass from agro-industrial activity provides cheap raw materials for the extraction of biopolymers, such as cellulose, hemicellulose, and lignin. Some advantages of these vegetal fibers are their natural abundance, low density, high specific stiffness, and biodegradability [8].

In recent years, with the rise of recycling policies, the paper industry has increased the production of paper from recycled cellulose fiber to produce cardboard packaging. These fibers have poorer physicochemical properties than the original cellulose fiber due to the hornification effect of the fiber. This effect is an alteration of the external layers of the cellulose that occurs in the drying process of paper and during its exposure to the environment, affecting the resistance properties of the paper. Mechanical beating is the most widely used technology at the industrial level, due to its cost and simplicity, to improve the properties of the recycled products that require it. However, even though the mechanical beating increases the specific surface area, swelling capacity, and mechanical properties of the fibers, there is also long-term structural damage and the creation of fines, thus reducing the drainage properties and life span of these products [9]. There are other technologies for dealing with the loss of mechanical properties of recycled products, such as the addition of virgin fiber, the addition of chemical products, or some more innovative ones, such as enzymatic refining or the addition of cellulose nanofibers [10]. Cellulose nanofibers present a high specific surface, compared to the original fiber, which allows a high adhesion capacity with adjacent fibers, acting as a link between fibers and favoring an increase in their mechanical properties [11].

Spain is the largest producer of vegetables in Europe, with a total of 12,629,447 tonnes in 2018, representing over 14% of total European production. In Spain, three of the most representative products are tomatoes, peppers, and eggplants which constitute 37.73%, 10.15%, and 1.89% of the vegetable production, respectively [12]. This production involves the generation of a high amount of waste that needs to be managed properly for its use in the production of high value-added products. The valorization of the lignocellulosic residues from these crops has been studied by several authors as a raw material to produce compost [13], biogas [14], particleboards [15], and cellulosic pulp for paperboard [16].

The aim of this work was to study the suitability of biomass residues from tomato, pepper, and eggplant as new sources for lignocellulosic nanofibers (LCNF) production and their application as a paperboard reinforcing agent. Cellulose pulps were obtained using an environmentally friendly process, energy-sustainable, simple, and with low-chemical reagent consumption. The obtained pulps were physicochemical characterized and used for LCNF production by mechanical and TEMPO-mediated oxidation pretreatment followed by high-pressure homogenization treatment. The LCNF obtained was submitted to a physical-chemical characterization and used to improve the mechanical properties during the paperboard formation and compared with industrial mechanical beating.

## 2. Results and Discussion

### 2.1. Chemical Composition of Raw Materials and Obtained Cellulosic Pulps

The first objective of this study is the production of cellulose pulps from horticultural plant residues (tomatoes, peppers, eggplants), which was accomplished by subjecting the raw materials to a soft conditions’ soda pulping process. This treatment has previously been successfully applied to produce cellulosic pulps with optimal characteristics for the isolation of lignocellulose nanofibers from herbaceous biomass [17]. The yields from the pulping process were 25.73%, 24.76%, and 20.50% for eggplant, pepper, and tomato plants, respectively.

**Figure 1:**
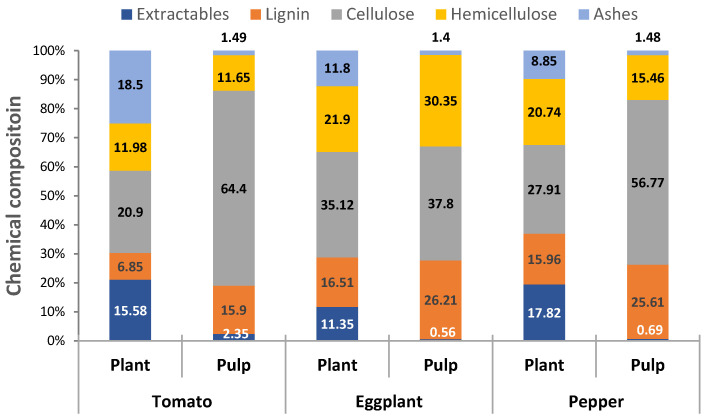
Chemical composition of horticultural plant residues and cellulosic pulps.

Figure 1 shows the differences in the chemical composition of the raw materials and that presented by the cellulosic pulps obtained. How the non-structural components (extractables and ashes) are reduced almost entirely after treatment is observed. The cellulose fraction was purified, especially in cellulosic pulps from tomatoes and pepper plants, increasing from 20.9% and 27.91% to 66.4% and 56.77%, respectively. In the case of eggplant, this important purification does not take place, however, and unlike the rest, an increase in the content of hemicellulose up to 30.35% was produced. The hemicellulose content is of special interest in the production of cellulose nanofibers through mechanical treatments acting as a barrier against the aggregation of the fibrillated microfibers. Chaker et al. determined that a hemicellulose content 25% produces a yield twice as high as that presented by fibers with 12% hemicellulose content [18]. The lignin content in the cellulosic pulp is high in comparison with other agri-residues used for lignocellulose nanofiber production [17,19,20,21]. Lignin acts as binding agent in the lignocellulosic matrix, promoting integrity and impeding its deconstruction. Despite its high lignin content, the pulping process may break ether and ester linkages between lignin and carbohydrates (cellulose and hemicellulose), allowing fiber defibrillation [22]. In addition, the presence of lignin in the fiber can exert an antioxidation action during nanofibrillation, preventing the union of links already broken during nanofibrillation treatment [23].

**Figure 2:**
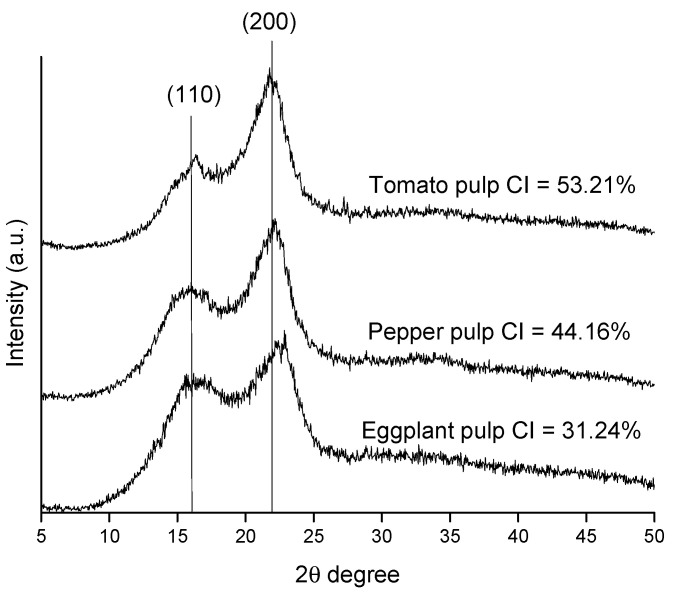
XRD patterns of horticultural residues pulps.

The crystallinity structure of the different cellulose pulps was analyzed by the X-ray diffraction technique (Figure 2). It is possible to observe two major reflection peaks at 2θ = 16.1° and 22.5°, corresponding to 110 and 200 typical reflection planes of cellulose I. The crystallinity index shows values of 31.24%, 44.16%, and 53.21% for eggplant, pepper, and tomato plants, respectively. The crystallinity index (CI) values shown by the cellulosic pulps are low compared to those shown by purified cellulose [20]. This is due to the presence of amorphous components, mainly lignin and hemicellulose, in fiber. These values coincided with the lower values of cellulose in fiber for eggplant and pepper pulp and, therefore, higher contents of hemicellulose and lignin.

### 2.2. Lignocellulose Nanofiber Characterization

In order to investigate the effect of the chemical composition of cellulosic pulps and of the different pretreatments on the production of lignocellulose nanofibers, the LCNF obtained were characterized in terms of nanofibrillation yield, cationic demand, carboxyl content, and morphology (Table 1). In general, it is observed that LCNF obtained by TEMPO-mediated oxidation presents a higher nanofibrillation yield (49–70%) compared to those obtained by mechanical pretreatment (18–33%). The nanofibrillation yields of LCNF obtained by mechanical pretreatment are similar to those presented by other cellulose nanofibers produced by the same process [24,25,26,27,28], however, those obtained by TEMPO-mediated oxidation show a slightly lower yield than those shown by other authors, which usually exceed 90% [29,30,31]. This behavior is also observed in the cationic demand where the TEMPO-oxidized LCNF present considerably higher values of cationic demand. This is mainly due to the greater specific surface (σ_LCNF_) that these nanofibers present in comparison with those obtained by means of mechanical pretreatment. This greater specific surface results in a greater exposure of the -OH and -COOH groups of the surface of the nanofibers, resulting in higher cationic demand. Regarding the carboxyl content, a slight increase is observed after catalytic oxidation. These values are very low in comparison with other nanofibers obtained by TEMPO-mediated oxidation that present carboxyl content values of 600–1000 μmols/g [26,29,30,31] This is due to the conversion of primary C6 alcohol groups from the surface of the crystalline regions of the cellulose into carboxyl groups produced during pretreatment. In this case, the low crystallinity, and the high presence of lignin (which may partially or totally consume the NaClO used as a catalytic reaction activator), means that this conversion is not as effective as for bleached pulps.

**Figure 3:**
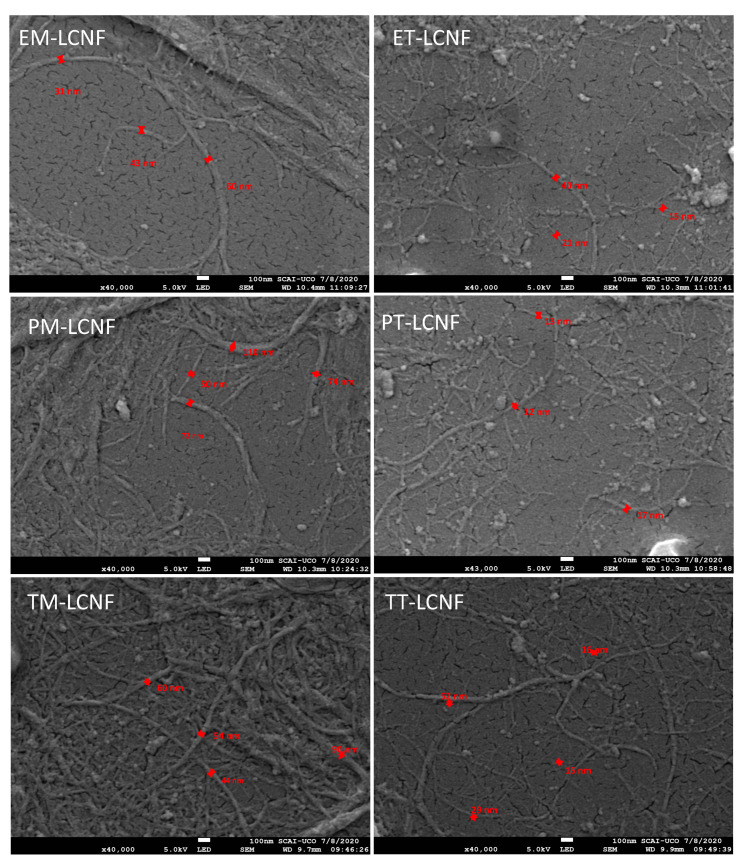
SEM microphotography of the different LCNF.

Regarding the morphology, some differences are observed between the different pretreatments. The diameters obtained vary significantly between the TEMPO-oxidized LCNF and those obtained by mechanical pretreatment. On the one hand, the nanofibers obtained by TEMPO-mediated oxidation show very similar diameter values (12–17 nm) despite the differences shown in the chemical composition. However, in LCNF obtained by mechanical pretreatment, large differences were observed between the different raw materials. It is observed that for LCNF obtained mechanically from eggplant residues, show much smaller diameter than the rest. This is due to their high hemicellulose content, which acts as a key component in the nanofibrillation process [18]. The namometric size of the LCNF was confirmed by direct observation by SEM (Figure 3). It is observed how significant differences exist between the different pretreatments, observing in the case of those obtained mechanically a large proportion of non-nanofibrillated macro/microfibers, as indicated by their low nanofibrillation yield compared to TEMPO-oxidized LCNF. Regarding the length of the lignocellulose nanofibers, a generalized decreased is observed after catalytic oxidation, showing a decrease of 60.07%, 55.94%, and 53.60% for the LCNF obtained from eggplant, pepper, and tomato plants. It is produced by the depolymerization and β-elimination of the cellulose amorphous regions into gluconic acid or cellulose-derived small fragments [32].

**Figure 4:**
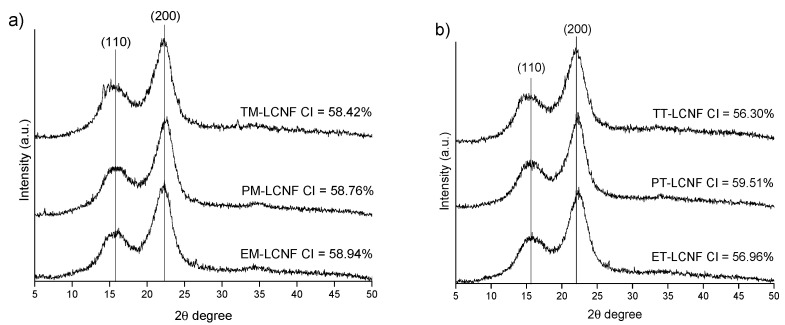
XRD patterns of LCNF obtained by mechanical (**a**) and TEMPO-oxidation (**b**) pretreatments.

Figure 4 shows the effect of the different pretreatments on the crystallinity of the lignocellulose nanofibers. In the different patterns crystalline reflection peaks are observed in the planes (110) and (200) as in the case of the cellulose pulps, indicating that LCNF also presents a crystalline structure typical of cellulose I. These observations suggest that the crystalline structure of the fiber is maintained after the different pretreatments and the nanofibrillation treatment. Both pretreatments present similar values for the different lignocellulose nanofibers (56%–60%), all higher than the initial values shown in the cellulose pulps. This may be due to the degradation of the amorphous regions by the action of both pretreatments, maintaining the crystalline regions of cellulose and increasing the crystallinity index of the samples [32].

**Figure 5:**
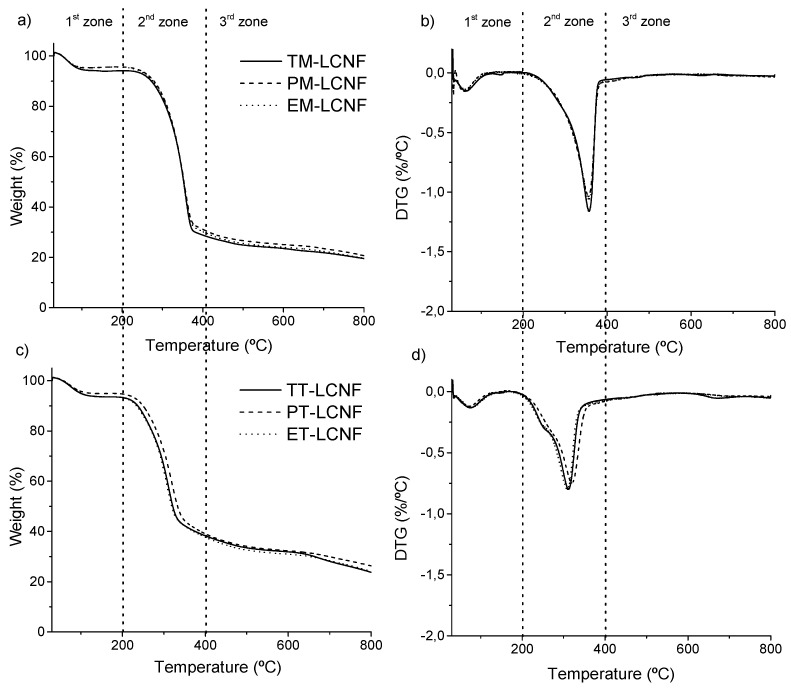
TGA and DTG curves of mechanical (**a,b**) and TEMPO-oxidation (**c,d**) LCNF.

The thermal stability of the cellulose nanofibers is also an important parameter to study their suitability in their final application. Figure. 5 shows the thermal behavior of the different cellulose nanofibers. The analysis of the different curves, identifies that the thermal decomposition is carried out in three zones, indicating the presence of distinct components decomposing at different temperatures. The first zone (up to 200 °C), shows a small decrease in the weight of samples related to the evaporation and removal of absorbed and bounded water in fiber [33,34,35]. The second zone (200–400 °C) corresponds to the active pyrolysis of the lignocellulosic components and is where the main degradation of the samples, including the maximum degradation temperature (T_max_) shown by the DTG curve. In the third zone (temperature above 400 °C) the passive pyrolysis of the lignocellulosic components takes places, where the low degradation ratio stands out and is identified with the lignin degradation and any carbonaceous matter decomposition [35,36].

The main peak observed in the DTG analysis (Figure 5b,d) shows the temperature where the thermal degradation is maximum, known as T_max_. The analysis shows that the cellulose nanofibers obtained by mechanical pretreatment show very similar values, being these 356.2 °C, 355.5 °C, and 357.5 °C for eggplant, pepper, and tomato plants. A similar behavior is observed for the lignocellulose nanofibers obtained by TEMPO-mediated oxidation, where the show a very similar value, being 308.8 °C, 317.8 °C, and 306.5 °C for eggplant, pepper and tomato, respectively. The lower stability shown by TEMPO-oxidized nanofibers is due to two main factors: i) The higher specific surface that results in a larger surface area exposed to heat; and ii) the introduction of carboxyl groups on the surface that produces a high number of free ends [24].

### 2.3. Lignocellulose Reinforcement on Recycled Paperboard

**Figure 6:**
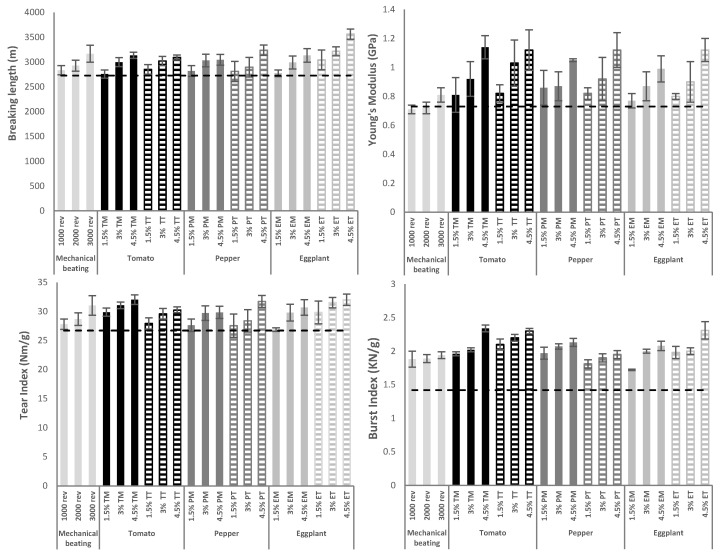
Evolution of the mechanical properties of recycled paperboard after different treatments.

The recycling process subjects the fibers to dispersion-drying cycles that reduce the binding capacity of the fibers, hornification process and, therefore, the mechanical properties shown by the final products. To correct this decrease, the industry uses different processes in order to increase the union between fibers, such as mechanical beating and the addition of chemical or virgin fiber [9]. The reinforcement effect on the recycled paperboard suspension of the lignocellulose nanofibers obtained in this work was compared with the effect produced by mechanical beating in order to analyze the suitability of the addition of LCNF as an alternative to mechanical beating. Figure 6 shows the evolution of the mechanical properties (breaking length, Young’s modulus, tear index, and burst index) of recycled paperboard after the different treatments. In a generalized way, it is observed how both treatments, mechanical beating and LCNF addition, produce an increase in the mechanical properties compared to the values obtained from the original recycled fiber (baseline). The mechanical properties shown by the original recycled fiber were 2726 m for breaking length, 0.73 GPa for the Young’s modulus, 26.71 Nm/g for the tear index, and 1.42 KN/g for the burst index. A linear increase is observed as the intensity of the mechanical refining of the amount of LCNF added increases, obtaining the highest values at the most severe conditions (3000 rev and 4.5% LCNF). The addition of LCNF produces a similar increase to the mechanical beating in breaking length and tear index parameters, however, it produces a more pronounced reinforcing effect on the Young’s modulus and burst index. The differences in the reinforcing effect were analyzed depending on the raw material used to produce lignocellulose nanofibers. In general terms, it is observed that the addition of LCNF produces a similar effect regardless of the raw material used. Regarding the pretreatment, it is observed that there are no major differences in the reinforcing effect between LCNF obtained by mechanical or TEMPO-mediated oxidation pretreatment. Although all LCNF produce a similar effect, the reinforcement produced by TEMPO-oxidized nanofibers from eggplant residue (ET) stands out. The addition of 4.5% ET produces an increase of 30.63%, 53.42% 19.88%, and 62.68%, compared to 16.18%, 11.00%, 16.21%, and 36.62% produced by mechanical beating for the breaking length, Young’s modulus, tear index, and burst index, respectively.

**Figure 7:**
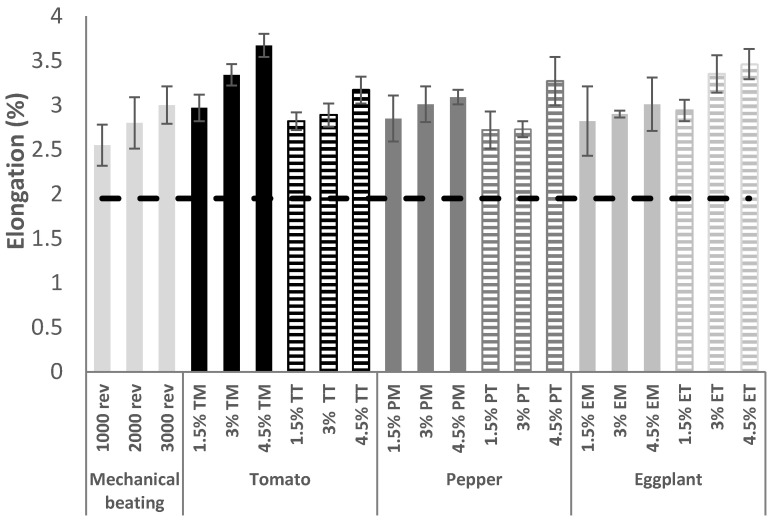
Evolution of the elongation at break of recycled paperboard after different treatments.

The increase in Elongation at break was also analyzed (Figure 7). For this parameter, as with the other mechanical properties, a linear increase is observed as the treatment becomes more severe, and the reinforcement effect is more effective with the addition of LCNF than with mechanical beating.

The increase in the mechanical properties due to the different treatments is given by: ( i) the increase in the specific surface area of fibers which facilitate the bonding with adjacent fibers produced by the mechanical beating, or (ii) the generation of a network embedded between the paperboard substrate fibers and the LCNF added, increasing the bonding capacity and facilitating the interfiber bonding [37].

The industrial paperboard presents a breaking length value of 5656 m. Considering that in the industrial papermaking process formation is produced in an isotropic way, it is necessary to perform a conversion of the isotropic-anisotropic values to compare them with those obtained in an anisotropic laboratory papersheet former. For this, the anisotropic ratio of 1.65 is considered, obtaining that the industrial cardboard shows a breaking length value of 3443 m [38]. According to the data obtained, under the conditions studied in this work, only the addition of 4.5% ET would reach this value (3561 m), fully correcting the loss of the mechanical properties during the recycling process. The use of LCNF, in addition to producing a greater reinforcing effect, would make it possible to increase the recycling cycles of the same fibers from three to 10 or more [37]. The low number of recycling cycles limit when using mechanical beating is due to the structural damages caused by the effect of shearing during the beating. Previous studies have confirmed that the use of this technology can be energy-efficient depending on the nanofibrillation treatment [38]. This phenomenon also explains the increase in density compared to untreated recycled paperboard, as well as the decrease in the porosity.

The influence of the different treatments on the evolution of the physical properties was also analyzed, as shown in Table 2. A decrease in thickness is observed as the severity of the different treatments increases. This is due to the greater bonding strength between the adjacent fibers, resulting in greater compaction of the fibrillar network [39]. These observations are more intense in the addition of LCNF compared to those shown by the mechanical beating treatment. This is due to the fact that in addition to the higher bonding strength, the nanometric size of the LCNF allowing them to fill the gaps between the fiber matrix, increasing the density and decreasing the porosity [39].

**Figure 8:**
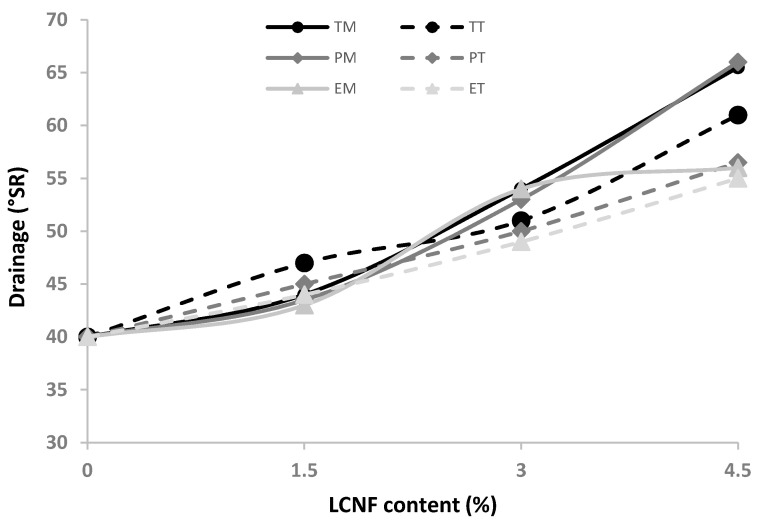
Evolution of drainability properties of cardboard suspension with different LCNF amount.

Figure 8 Shows the evolution of the drainage properties of the paperboard slurries after the addition of the different LCNF. As expected, the addition of lignocellulose nanofibers, due to their specific surface area and their hydrophilic nature, results in a high water-holding capacity, thus increasing the viscosity of the suspension and decreasing the drainage capacity of the slurries. The mechanical beating treatment, due to the large generation of fines during the process, also produces a decrease in the drainage capacity showing a drainage degree of 47°SR, 49°SR, and 54°SR for 1000 rev, 2000 rev, and 3000 rev, respectively. These values are lower compared to those obtained by the addition of LCNF; however, this property is not a key parameter when evaluating the suitability of this technology for use in the recycling paperboard industry since this property can be corrected by the combination of nanofibers and electrolytes [40].

## 3. Materials and Methods

### 3.1. Materials

The greenhouse residues of tomato (*Solanum lycopersicum*), pepper (*Capsicum annuum*), and eggplant (*Solanum melongena*) were used in this work, which corresponded to the entire plants being completely uprooted from the ground (this allows for planting seeds for a new crop), after harvesting the fruits. These greenhouse residues were provided by Aguadulce Cooperative from Aguadulce, Almería (Spain). First, vegetable materials were cleaned and dried at room temperature. Then, they were cut into pieces of 0.1–1 cm and stored in plastic bags for preservation until use. As the paperboard substrate, a suspension of cellulose fiber from recycled paper and cardboard was used. This was not subjected to any mechanical refining, or the addition of chemicals or virgin fiber cellulose. This suspension was kindly supplied by the firm Smurfit Kappa Container 100 S.L. (Mengíbar, Jaén, Spain).

### 3.2. Soda Pulping

The soda pulping process is carried out to make the cellulose fiber more accessible for the pretreatments performed, also to improve the efficiency of the nanofibrillation process. The raw materials were pulped, according to proper conditions of Specel^®^ process, in a 15 L batch reactor (Metrotec S.A., Lezo, Spain) with 7% NaOH over dried material (o.d.m) at 100 °C for 150 min and a liquid/solid ratio 10/1. The cellulosic pulp was dispersed in a pulp disintegrator (Metrotec S.A., Lezo, Spain) at 1200 rpm for 30 min, and this disintegrated pulp was passed through a Sprout-Bauer beater (Combustion Engineering, Vienna, Austria). The pulp suspension was separated by sieving through a 0.14 mm mesh to retain uncooked material [17,41]

### 3.3. Pulp Characterization

The chemical characterization of raw materials and cellulosic pulps was done in terms of their content in ethanol extractables, ash, holocellulose, lignin, and α-cellulose, according to TAPPI standards T-204, T-211, T-222, T-203os61, and T-9m54, respectively. In addition, the pulping yield was calculated according to Equation (1):

(1) $$Yield\;\left(\%\right)=\frac{W_{1}}{W_{0}}\cdot 100$$

where W_1_ corresponds to the dry weight of the samples after removing the uncooked material and W_0_ corresponds to the initial dry weight of raw materials.

### 3.4. Lignocellulose Nanofibers (LCNF) Production

Lignocellulose nanofibers were produced from the obtained cellulosic pulp by using two different pretreatments: mechanical and TEMPO-mediated oxidation, followed by high-pressure homogenization treatment.

#### 3.4.1. Mechanical Pretreatment

The cellulosic pulp was refined in PFI beater (Metrotec S.A., Lezo, Spain), according to ISO 5264-2:2002, until obtaining the drainage degree (°SR) closest to 90°. For all samples, 30,000 revolutions were required to obtain the above °SR value.

#### 3.4.2. TEMPO-Mediated Oxidation Pretreatment

The cellulosic fibers were subjected to TEMPO-mediated oxidation pretreatment following the methodology described by Besbes et al. [42]. An amount of NaClO suspension (equivalent at 5 mmols/g cellulose) was added with continuous stirring at room temperature. Then, the pH value was maintained at 10.2 with the addition of 0.5 M NaOH until no pH decrease was observed. Finally, the fibers were filtered and washed several times with distilled water.

#### 3.4.3. High-Pressure Homogenization

Both pretreated fibers suspensions (1% concentration) were passed through a high-pressure homogenizer Panda GEA 2K (GEA Niro, Parma, Italy) as nanofibrillation treatment. The high-pressure homogenization was made through 10 cycles (four cycles at 300 bar, three cycles at 600 bar, and three cycles at 900 bar) [24]. This increasing pressure avoids problems of clogging in the equipment because of the initial fiber size. Table 3 shows the codification used for the different samples.

### 3.5. LCNF Characterization

To determine the nanofibrillation yield, a 0.1% LCNF suspension was centrifuged at 10,000 rpm for 12 min. The precipitated fraction or non-nanofibrillar material was separated from the nanofibrillar material, and it was dried at 100 °C for 24 h [42].

The cationic demand (CD) was determined through the adaptation of the methodology followed by Carrasco et al. [43]. First, 0.20 dried grams of LCNF were diluted in distilled water until 200 g. When the suspension was homogenized, 10 mL of this solution was mixed with 25 mL of a cationic polymer (poly-DADMAC). The suspension was centrifuged at 4000 rpm for 90 min. Then, 10 mL of supernatant was introduced to a Mütek PCD 05 particle charge detector and back-titrated with the anionic polymer (PesNa) until the detector indicated zero conductivity. The CD was determinate according to Equation (2):

(2) $$CD=\frac{\left(CpolyD.\;VpolyD\right).\;\left(CPesNa.\;VPesNa\right)}{m}$$

where C_polyD_ and V_polyD_ correspond to Poly-DADMAC concentration and volume, respectively; C_PesNa_ and V_PesNa_ correspond to PesNa concentration and volume, respectively; and m is the weight of the dried product (g).

According to the methodology of Besbes et al. [42], the carboxyl content (CC) was determined by conductometric titration. First, 50–100 mg of dry fiber was suspended in 15 mL of HCl (0.01 M) to exchange the Na^+^ cations attached to COOH groups by H^+^ ions. Then, the suspensions were titrated with NaOH (0.01 M), adding 0.1 mL of NaOH to the sample suspensions and recording the conductivity, observing a reduction, stabilization and increase in the conductivity. The CC was determined according to the Equation (3):

(3) $$CC=\frac{\left(V_{2}-V_{1}\right)\cdot \left[NaOH\right]}{m}$$

where V_2_ and V_1_ are the equivalent volumes of added NaOH solution; [NaOH] corresponds to NaOH solution concentration and m stands for the weight of the dried product (g).

Considering the assumptions and methodology described by Carrasco et al. [44], the obtained values of the cationic demand and carboxyl content were used for the theoretical estimation of the specific surface (σ_LCNF_) and diameter of the LCNF.

The cationic polymer (Poly-DADMAC) used for the cationic demand determination interacts with cellulose fibers through surface adsorption mechanisms. The Poly-DADMAC has a specific area of 4.87 × 10^17^ nm^2^/µeq·g; based on this value, the specific surfaces of the LCNF obtained were calculated according to Equation (4) [39]:

(4) $$\sigma_{LCNF}=\left(CD-CC\right)\cdot \sigma_{Poly-DADMAC}$$

where σ_LCNF_ is the specific surface of LCNF (nm^2^/g); CD is the cationic demand (µeq/g); CC is the carboxyl rate (µmols/g); and σ_Poly-DADMAC_ corresponds to the specific surface of this cationic polymer (nm^2^/µeq).

Assuming the cylindrical geometry of the LCNF, the value of the specific surface was used to determine its diameter.

All measurements were made in triplicate and mean, and standard deviations were calculated.

### 3.6. Polymerization Degree and Length

The intrinsic viscosity (η) was obtained according to UNE-57-039-92 and used for the calculation of the degree of polymerization. The degree of polymerization (DP) was calculated using Equations (5) and Equation (6) [45]:

(5) $$DP\;\left(<950\right):DP=\frac{\eta_{s}}{0.42}$$

(6) $$DP\left(>950\right):DP^{0.76}=\frac{\eta_{s}}{2.28}\;$$

The length of the nanofibers was calculated from the polymerization degree (DP) using the Equation (7) proposed by Shinoda et al. [46]:

(7) Length (nm)=4.286·DP−757

### 3.7. Spectroscopy Analysis

Fourier transform infrared spectroscopy (FTIR) analysis was applied to determine possible changes in the structure, chemical composition, and functional groups during LCNF isolation processes. A Spectrum Two FT-IR Spectrometer (Perkin-Elmer, Massachusetts, USA) was used in the range of 450–4000 cm^−1^ with a resolution of 4 cm^−1^_,_ collecting a total of 40 scans per sample.

### 3.8. X-ray Diffraction (XRD) Analysis

XRD was used to study the crystal structures of the cellulosic pulps and LCNF. This analysis was performed using a Bruker D8 Discover (Bruker Corporation, Massachusetts, USA) with a monochromatic source CuKα1 over an angular range of 5–50° at a scan speed of 1.56°/min. The Segal method was used to calculate the crystallinity index (CI)(Equation (8)) [47]:

(8) $$CI\left(\%\right)=\left(\frac{I_{200}-I_{am}}{I_{200}}\right)\cdot 100$$

where I_200_ is the diffraction peak intensity at 2θ = 22.5° of the crystalline cellulose regions, and I_am_, is the intensity minimum between two diffraction peaks (2θ = 16.5° and 22.5°) of the amorphous cellulose region.

### 3.9. Thermogravimetric Analysis

Thermogravimetric analysis (TGA) was performed to evaluate the thermal stability of the LCNF. The derivate thermogravimetric (DTG) was used to analyze the maximum degradation rate (T_max_). The analysis was carried out using a Mettler Toledo TGA/DSC 1 (Mettler Toledo, Ohio, USA). The samples were taken from room temperature to 800 °C with a heating rate of 10 °C/min and a 50 mL/min nitrogen gas flow rate.

### 3.10. Reinforcement of Industrial Pulp

LCNF was used as a reinforcing agent on a paperboard industrial pulp (Smurfit Kappa) and compared its effect with that produced by mechanical beating. The industrial cellulose pulp was submitted at different mechanical refining intensities (1000 rev, 2000 rev, and 3000 rev). Lignocellulose nanofibers (mechanical and TEMPO) of tomato, pepper, and eggplant were added at 1.5%, 3%, and 4.5% (o.d.m.) to the industrial cellulose suspension. The process started disintegrating 30 g dry weight pulp for 30 min. Then, LCNF was added in the established proportion and disintegrated for 1 h. After that, 0.5% (*w*/*w*) cationic starch (Vector SC 20157) and 0.8% (*w*/*w*) colloidal silica (LUDOX^®^ HS-40) were added to improve the retention of LCNF by improving the bonding of the fibers, based on the dry weight of the pulp and LCNF and kept in constant mechanical agitation. The papersheet formation was carried out in a sheet former ENJO-F-39.71 (Metrotec S.A., Lezo, Spain) according to TAPPI T205ps-95. Before the mechanical testing, the papersheets were conditioned at 25 °C and 50% relative humidity for 48 h. The mechanical characterization was performed using an Instron universal testing machine (Lloyd Instruments, Bognor Regis, United Kingdom) provided with 1 kN load cell, in terms of breaking length and tensile index, elongation, Burst index, and tear index, according to TAPPI standard (T-494-om96, T-494, T-403-om97 and T-414-om98, respectively). The physical properties of the papersheets were analyzed for their thickness, density, and porosity. The thickness was determined according to the standard ISO 534. The density was calculated from the weight of the sheets and their dimensions. The porosity of the sheets was calculated using Equation (9):

(9) $$Porosity\;\left(\%\right)=100\cdot \left(1-\frac{\rho_{sample}}{\rho_{cellulose}}\right)$$

where *ρ_sample_* is the density of the sheet, and *ρ_cellulose_* is the density of cellulose, assumed as 1.5 g/cm^3^.

## 4. Conclusions

Horticultural plant residues, as lignocellulosic source for the isolation of lignocellulose nanofibres, were analyzed. The cellulosic pulps obtained were subjected to two different pretreatments, mechanical and TEMPO-mediated oxidation, and a subsequent high-pressure homogenization process for lignocellulose nanofiber isolation. The different LCNF were added as a reinforcing agent on recycled paperboard and compared with the improvement produced by industrial mechanical beating. The addition of 4.5% TEMPO-oxidized LCNF from eggplant residues produces the greater increase in comparison with the other LCNF. It produces an increase of 30.63%, 53.42%, 19.88%, and 62.68%, compared to 16.18%, 11.00%, 16.21%, and 36.62% produced by mechanical beating for breaking length, Young’s modulus, tear index, and burst index, respectively. The use of LCNF produces a decrease in the papersheet thickness and porosity, and an increase in the density. This is due to the greater bonding strength between the adjacent fibers and the gap filling resulting in greater compaction of the fiber matrix. The use of LCNF in the paperboard recycling process as an alternative to mechanical beating produces a greater reinforcing effect and would make it possible to increase the recycling cycles of same fibers from three to 10 or more.

## Figures and Tables

**Table 1:** Characterization of the different lignocellulose nanofibers.

LCNF Sample	η (%)	CD (μeq/g)	CC (μmols/g)	σ_LCNF_ (m^2^/g)	Diameter (nm)	Length (nm)
TM-LCNF	17.81 ± 2.59	298.39 ± 48.30	247.35 ± 6.14	24.86	112	5440
PM-LCNF	18.34 ± 3.37	166.46 ± 0.00	148.71 ± 1.66	8.64	278	4317
EM-LCNF	32.61 ± 3.48	248.30± 10.89	127.25 ± 3.99	58.95	42	5132
TT-LCNF	48.77 ± 1.30	707.86 ± 18.54	299.96 ± 48.76	198.65	12	2524
PT-LCNF	69.66 ± 6.11	513.37 ± 37.23	205.81 ± 5.86	148.78	17	1902
ET-LCNF	66.39 ± 1.52	563.38 ± 37.06	186.61 ± 63.78	183.48	14	2049

η: nanofibrillation yield; CD: Cationic demand; CC: Carboxyl content; σ_LCNF_: Specific surface area of LCNF.

**Table 2:** Evolution of the physical properties of recycled paperboard after different treatments.

Treatment	Sample	Thickness (μm)	Density (g/cm^3^)	Porosity (%)
	Recycled paperboard	150.3 ± 2.9	0.36 ± 0.01	75.55 ± 0.79
Mechanical beating	1000 rev	147.8 ± 6.5	0.37 ± 0.01	75.10 ± 1.01
	2000 rev	147.2 ± 8.0	0.38 ± 0.02	74.93 ± 1.49
	3000 rev	146.8 ± 6.6	0.38 ± 0.01	74.75 ± 0.91
Tomato LCNF	1.5% TM	139.9 ± 4.2	0.39 ± 0.01	73.72 ± 0.93
	3% TM	136.6 ± 3.8	0.40 ± 0.02	73.06 ± 1.61
	4.5% TM	134.9 ± 2.7	0.41 ± 0.02	72.72 ± 1.38
	1.5% TT	142.4 ± 5.4	0.39 ± 0.01	74.53 ± 0.57
	3% TT	142.6 ± 1.9	0.39 ± 0.03	74.23 ± 0.74
	4.5% TT	138.4 ± 4.3	0.40 ± 0.02	73.38 ± 1.63
Pepper LCNF	1.5% PM	136.0 ± 4.9	0.40 ± 0.01	72.94 ± 1.34
	3% PM	134.2 ± 7.6	0.41 ± 0.02	72.82 ± 0.69
	4.5% PM	133.1 ± 5.6	0.41 ± 0.02	72.78 ± 0.72
	1.5% PT	147.5 ± 3.0	0.38 ± 0.02	75.08 ± 1.42
	3% PT	147.8 ± 2.1	0.38 ± 0.03	74.86 ± 1.06
	4.5% PT	147.4 ± 5.3	0.39 ± 0.01	74.04 ± 0.36
Eggplant LCNF	1.5% EM	146.0 ± 3.9	0.38 ± 0.01	74.91 ± 0.78
	3% EM	145.3 ± 4.5	0.39 ± 0.02	74.00 ± 1.83
	4.5% EM	138.7 ± 9.2	0.40 ± 0.02	73.43 ± 1.54
	1.5% ET	143.7 ± 8.1	0.38 ± 0.01	74.94 ± 0.83
	3% ET	146.7 ± 4.4	0.38 ± 0.02	74.51 ± 1.23
	4.5% ET	144.5 ± 6.1	0.39 ± 0.01	74.37 ± 0.66

**Table 3:** Codification of the different lignocellulose nanofibers.

Raw Material	Pretreatment	Treatment	Codification
Tomato	Mechanical	High-pressure homogenization	TM-LCNF
	TEMPO-mediated oxidation		TT-LCNF
Pepper	Mechanical		PM-LCNF
	TEMPO-mediated oxidation		PT-LCNF
Eggplant	Mechanical		EM-LCNF
	TEMPO-mediated oxidation		ET-LCNF
